# Provision of intensive care to severely ill pregnant women is associated with reduced mortality: Results from the WHO Multicountry Survey on Maternal and Newborn Health

**DOI:** 10.1002/ijgo.13241

**Published:** 2020-07-12

**Authors:** Fabiano M. Soares, Rodolfo C. Pacagnella, Özge Tunçalp, José G. Cecatti, Joshua P. Vogel, Ganchimeg Togoobaatar, Joao P. Souza

**Affiliations:** ^1^ Department of Obstetrics and Gynecology School of Medical Sciences University of Campinas (UNICAMP) Campinas Brazil; ^2^ UNDP/UNFPA/UNICEF/WHO/World Bank Special Programme of Research Development and Research Training in Human Reproduction (HRP) Department of Sexual and Reproductive Health and Research WHO Geneva Switzerland; ^3^ Department of Global Health Nursing Faculty of Medicine University of Tsukuba Ibaraki Japan; ^4^ Department of Social Medicine (Public and Family Health) Ribeirao Preto Medical School University of Sao Paulo Sao Paulo Brazil

**Keywords:** Cross‐sectional study, Intensive Care Unit, Maternal morbidity, Maternal mortality, Maternal near‐miss, Severity classification

## Abstract

**Objective:**

To estimate the impact of the use of Intensive Care Units (ICU) in maternal mortality.

**Methods:**

A secondary analysis of the WHO Multicountry Survey on Maternal and Newborn Health, a multicenter cross‐sectional study conducted in maternity hospitals in 29 countries. Women who had severe maternal outcome (maternal death or maternal near‐miss) and the availability and use of ICU beds were included. The women were categorized according to availability of ICU, and multivariate logistic regression analyses were performed to determine the risk of maternal death. To rate the severity of complications, the Maternal Severity Score (MSS) and the Maternal Severity Index (MSI) were used.

**Results:**

Of 314 623 women observed, 24 396 had severe complications. Of those, 16 981 (69.6%) were in facilities with ICUs; 1573 women were admitted to ICUs (6.4% of women with maternal complications and 0.5% of total). There is a significant protective effect for maternal mortality for patients with more severe conditions using ICUs (odds ratio 0.16, 95% confidence interval 0.07–0.33).

**Conclusion:**

The use of ICU was associated with significantly reduced odds of maternal death in obstetric patients with severe clinical conditions. The availability and appropriate use of good‐quality ICUs are therefore crucial to reduce maternal mortality.

## Abstract 2

The use of Intensive Care Unit (ICU) beds by women with severe clinical conditions may interfere with maternal mortality.

## 1 INTRODUCTION

The reduction of maternal mortality is a global public health priority, not only because the majority of maternal deaths are avoidable, but also because maternal mortality represents broader gender and social inequities across and within countries.^1^, ^2^, ^3^ Substantial progress has been achieved in reducing maternal mortality, but high rates of maternal mortality persist in many countries.^4^, ^5^, ^6^, ^7^

One challenge in reducing maternal deaths is limited access to obstetric emergency services; however, one strategy to reduce maternal mortality is to improve emergency care for maternal conditions and include the availability of Intensive Care Units (ICU) for these patients. An ICU is generally defined as a self‐contained and equipped area of a hospital complex with special staff dedicated to managing and monitoring patients with life‐threatening conditions. The specific definitions of staff and equipment may vary depending on the particular healthcare system and availability of resources.^8^

The overall rate of maternal mortality in ICUs varies widely, in the range of 3%–21%, being significantly higher in lower‐income countries.^9^, ^10^ However, approximately 50% of these maternal deaths are due to modifiable factors or treatable conditions, such as complications of pre‐eclampsia.^9^ Although there has been an increasing number of ICU beds, and new technologies have been developed and used in ICUs, these units are not adequately used for critical obstetric conditions, even in high‐income countries.^11^

Few studies have assessed whether ICUs have an effect in reducing maternal mortality. There is a need for studies to explore and understand the use of ICUs for obstetric complications, especially in low‐ and middle‐income countries where health resources are scarce and the costs of ICUs are high.^12^ With increasing healthcare expenditures, it is necessary to show evidence of effectiveness to drive investment.^13^ The aim of the present study was to evaluate the impact of the availability and use of ICUs in the reduction of maternal mortality among women with severe complications related to pregnancy and childbirth.

## 2 MATERIALS AND METHODS

This is a secondary analysis of the WHO Multicountry Survey on Maternal and Newborn Health (WHOMCS), a multicenter cross‐sectional study conducted in 29 countries. Detailed information on the study methods is provided elsewhere.^14^ In summary, the WHOMCS collected data from 359 health facilities between May 1, 2010 and December 31, 2011. To select countries and health facilities, a stratified, multistage random sampling strategy was used (probability proportional to population size or number of births).

From these, up to seven institutions with over 1000 deliveries per year and capacity for cesarean delivery were randomly selected. All women giving birth and all women with a severe maternal outcome (SMO; death or near‐miss) associated with pregnancy or childbirth in participating institutions during the data collection period were included. This included women that had a SMO due to an abortion or ectopic pregnancy (i.e. without delivery). Data were captured on all eligible participants, from presentation to the institution until discharge or the seventh day postpartum, whichever came first. Although official definitions of maternal mortality and morbidity go up to 42 days postpartum, due to the methodological aspects of data collection, the WHOMCS Steering Group elected to use up to 7 days postpartum as a pragmatic period for the study design. SMO was defined as a woman who had a maternal death or maternal near‐miss up to 7 days after giving birth or having an abortion, irrespective of gestational age or delivery status. According to Say et al.,^15^ a maternal near‐miss was defined as a woman who survived a life‐threatening condition with organ dysfunction.

Data regarding clinical condition and outcomes were collected in a paper data collection form and then entered into a web‐based electronic database for analysis.

Data on the characteristics of each facility (including information on the size, capacity, location, and available resources) and their ability to identify and manage severe complications were collected. For the characterization of participating institutions, the Facility Capacity Index (FCI) was used, which assigned a composite score to each institution based on its capacity to identify and manage complications.^16^ Basic services, medical services, emergency obstetric services, laboratory tests, the standards of hospital practices, and human resources were considered for the composition of this index. The FCI was calculated as a continuous index.^17^

The present study focused only on women who had severe complications during pregnancy and the postpartum period. The availability and use of ICU were analyzed. Maternal complications were classified as hemorrhagic, hypertensive, infectious, abortion and ectopic pregnancy, and others. In addition, some descriptive variables of the population, such as age, education, marital status, and number of pregnancies, were also used in the analysis.

The 2012 Human Development Index (HDI) ranking was used as a proxy variable for the differences between countries.^18^ For rating the severity of maternal complications, the Maternal Severity Score (MSS) and the Maternal Severity Index (MSI) were used.^4^ The MSS is calculated by the sum of each severity marker (from the WHO near‐miss criteria) presented in a woman’s clinical condition and is in the range of 0–25. The MSI is calculated based on the MSS and expresses the risk of death as a percentage.^4^ The MSS identifies the occurrence of organ dysfunction at any point in time, even after hospital admission. It is important to emphasize that the MSS is based on markers of organ dysfunction and shows a correlation between the number of markers of organ dysfunction and mortality, and this can correlate with clinical severity as the number of markers of organ dysfunction is proportional to mortality.

Following the definitions in the WHO near‐miss approach for maternal health,^19^ indicators of ICU use were calculated: rate of admission to an ICU among all participants; admission rate to an ICU among women with severe maternal outcomes; rate of SMO among women admitted to an ICU; and proportion of maternal deaths observed without admission to an ICU.

Exploratory data analysis was conducted to examine the data for frequencies, distributions, and missing data by availability of ICU and use status. This variable was grouped under the following categories: (1) no ICU available; (2) ICU available but no admission to ICU; and (3) ICU available and admission to ICU. Pearson χ^2^ trend tests were conducted, taking into account the clustering effect of the survey design, to compare the distribution of women experiencing complications according to availability of and admission to an ICU.

Multivariate logistic regression analyses were conducted to assess the association between availability and use of an ICU among severely ill women in facilities and maternal mortality using three different models. Known variables associated with adverse maternal outcomes were selected, as well as proxies of socioeconomic status a priori. All models used maternal mortality as the outcome, and reference groups for each of the variables included in the model have been identified based on the assessment of the distribution. In these models, variables influencing the relationship between access to and use of an ICU and use and maternal mortality in facilities were progressively examined and controlled for. The first group of variables was individual and facility level including variables on maternal age, marital status, level of educational, number of pregnancies, and FCI. Then in Model 2, the MSS was included in the model followed by the HDI (Model 3). All the variables included in the model were assessed for collinearity and the models were adjusted for non‐independence (clustering) at the facility level.

Statistical analyses were conducted using Stata/MP version 13.0 (Stata Corp LP). A *P* value less than 0.05 was considered statistically significant.

The present study was approved by the WHO ethical review committee and the relevant ethical clearance mechanisms in all countries. Written consent from individual participants was not required.

## 3 RESULTS

**Figure 1:**
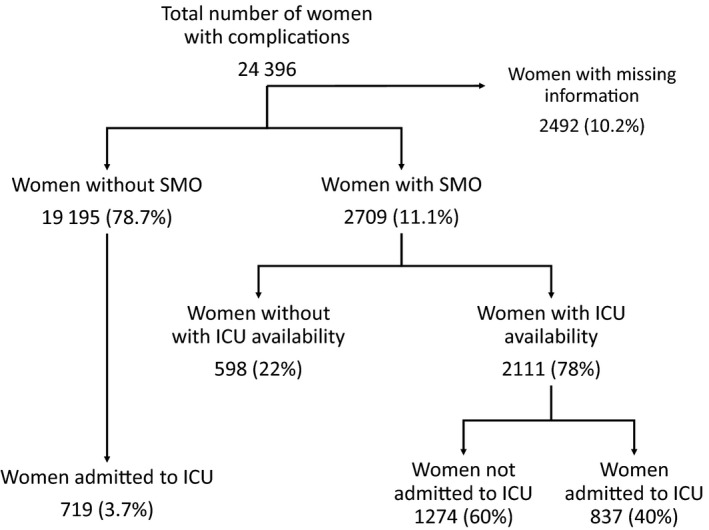
Distribution of women in the study according to access to and use of an ICU. Abbreviations: ICU, Intensive Care Unit; SMO, severe maternal outcome.

Of the 314 623 women included in the WHOMCS, 24 396 had severe complications and were included in the present study. Among women with any complication, 16 981 (69.6%) were in facilities with an available ICU. A total of 1573 women were admitted to an ICU (6.4% of women with maternal complications and 0.5% of all women). A total of 2492 (10.2%) women were excluded from the analysis due to missing information on access and/or use of an ICU (Fig. 1).

**Figure 2:**
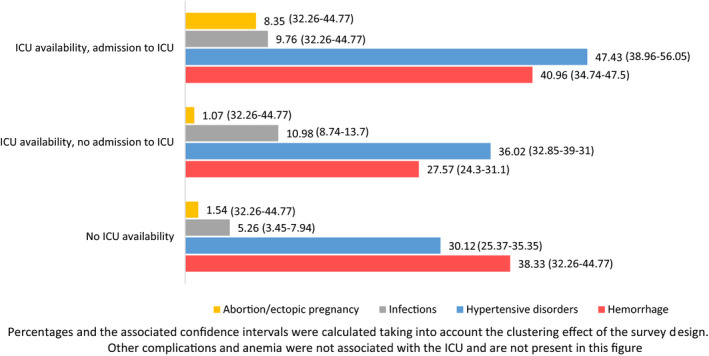
Distribution of women experiencing complications according to availability of and admission to an ICU. Abbreviations: ICU, Intensive Care Unit.

It was observed that 0.5% of all women in the study were admitted to an ICU (Table 1). Among those identified as SMO, 30.9% were admitted to an ICU. However, the frequency of SMO among patients who used an ICU was 53.8%. There were 424 maternal deaths. Most maternal deaths (70%) occurred in women who were not admitted to an ICU (Fig. 2).

**Table 1:** Use of indicators of intensive care.^a^

Intensive care unit use	Units
Total number of women participating in the study	314 623 (100)
Total number of women in this analysis	24 396 (7.8)
Rate of admission to ICU among women participating in the study	1573 (0.5)
Rate of admission to ICU among women with SMO	837 (30.9)
SMO rate among women admitted to an ICU	837 (53.8)
Proportion of maternal deaths without admission to an ICU	424 (70)

Abbreviations: ICU, Intensive Care Unit; SMO, severe maternal outcome.

^a^ Values are given as number (percentage).

The majority of the study population was aged 20–34 years. Higher levels of education were less frequent in the group with no ICU availability (20.7% and 15.1%) and in the group with ICU admission (22.1% and 8.5%). Women admitted to an ICU were less educated (27.5%). Primiparity was more frequent among those admitted to an ICU (34%).

Institutions with ICU availability showed higher values of FCI (53.5%). Patients admitted to an ICU had higher mean values of MSS and MSI than the other groups (Table 2).

**Table 2:** Characteristics of women with complications by status of ICU access (n=21 904).^a^

Women and facility characteristics	Status of ICU access			
	No ICU available	ICU available, no admission to ICU	ICU available, admission to ICU	Total
Age (years)				
<20	522 (10.6)	1648 (10.7)	169 (10.9)	2339 (10.7)
20–34	3580 (72.7)	11 156 (72.3)	1006 (64.6)	15 742 (71.9)
≥35	807 (16.4)	2604 (16.9)	376 (24.2)	3787 (17.3)
Missing	14 (0.3)	17 (0.1)	5 (0.3)	36 (0.2)
Marital status				
Without a partner	507 (10.3)	1582 (10.3)	147 (9.4)	2236 (10.2)
With a partner	4255 (86.4)	13 773 (89.3)	1389 (89.3)	19 417 (88.6)
Missing	161 (3.3)	70 (0.4)	20 (1.3)	251 (1.2)
Years of schooling (UNESCO)				
No education	959 (19.5)	2044 (13.3)	428 (27.5)	3431 (15.7)
Primary education	625 (12.7)	2112 (13.7)	199 (12.8)	2936 (13.4)
Lower secondary	951 (19.3)	2873 (18.6)	278 (17.9)	4102 (18.7)
Upper secondary	1020 (20.7)	4611 (29.9)	344 (22.1)	5975 (27.3)
Tertiary	743 (15.1)	2669 (17.3)	133 (8.5)	3545 (16.2)
Missing	625 (12.7)	1116 (7.2)	174 (11.2)	1915 (8.7)
Number of pregnancies^b^				
1	1678 (34.1)	5692 (36.9)	529 (34.0)	7899 (36.1)
2–3	1993 (40.5)	6248 (40.5)	519 (33.3)	8760 (40.0)
>3	1247 (25.3)	3477 (22.5)	505 (32.5)	5229 (23.8)
Missing	5 (0.1)	8 (0.1)	3 (0.2)	16 (0.1)
*SMO*	598 (22.1)	1274 (47.0)	837 (30.9)	2709 (100)
*FCI*	53.5 ± 18.6	58.1 ± 12.3	54.8 ± 4.2	56.8 ± 13.7
*MSS*	0.20 ± 0.7	0.16 ± 0.8	1.76 ± 2.8	0.28 ± 1.1
*MSI*	0.004 ± 0.04	0.005 ± 0.05	0.07 ± 0.19	0.009 ± 0.07

Abbreviations: FCI, Facility Capacity Index; ICU, Intensive Care Unit; MSI, Maternal Severity Index; MSS, Maternal Severity Score; SD, standard deviation.

^a^ Values are given as number (percentage) or mean ± SD.

^b^ Including the current pregnancy.

In the multivariate analysis, different sets of variables were used to identify the association between use of an ICU and the odds of dying among women with severe maternal morbidity (Table 3). In Model 1, controlling for individual and facility characteristics, higher level of education (odds ratio [OR] 0.76, 95% confidence interval [CI] 0.65–0.90), and number of pregnancies (OR 0.75, 95% CI 0.61–0.91) were significantly associated with reduction in the odds of maternal death, while the use of an ICU was not significantly associated with maternal death (OR 1.19, 95% CI 0.63–2.26). In Model 2 (including the MSS as predictor), higher level of education and admission to an ICU had an OR for maternal death of 0.69 (95% CI 0.57–0.84) and 0.16 (95% CI 0.07–0.33), respectively, while the degree of severity had an OR of 1.94 (95% CI 1.71–2.19) for maternal death. When the HDI was included in Model 3, the results showed that while the HDI was significantly associated with maternal death, it did not change the association between admission to an ICU and maternal mortality (0.18, 95% CI 0.08–0.41). When the models were run separately for hemorrhage, hypertension, and infection, the association between admission to an ICU and maternal mortality continued to hold and got stronger for hypertensive disorders (0.08, 95% CI 0.02–0.29). However, the sample size for these sub‐analyses was smaller, especially for infection (n=301); the analytical sample was 678 for hypertension and 920 for hemorrhage.

**Table 3:** The association between ICU availability and admission and maternal death among women who experience SMO (n=2404^a^).

Women and facility characteristics	OR (95% CI)	Model 1^b^	Model 2	Model 3
		Adjusted OR (95% CI)	Adjusted OR (95% CI)	Adjusted OR (95% CI)
ICU access among women with SMO				
With no ICU availability	Ref	Ref	Ref	Ref
With ICU availability, no admission	1.09 (0.63–1.88)	1.19 (0.63–2.26)	0.79 (0.43–1.45)	0.76 (0.39–1.48)
With ICU availability, admission	1.01 (0.55–1.86)	1.06 (0.50–2.27)	**0.16 (0.07–0.33)**	**0.18 (0.08–0.41)**
Individual and facility characteristics				
Age	—	1.23 (0.99–1.52)	1.18 (0.89–1.56)	1.22 (0.91–1.64)
Marital status (with a partner)	—	0.69 (0.42–1.12)	0.68 (0.38–1.23)	**0.52 (0.28–0.95)**
Level of education (higher)	—	**0.76 (0.65–0.90)**	**0.69 (0.57–0.84)**	**0.76 (0.63–0.92)**
Number of pregnancies (higher)	—	**0.75 (0.61–0.91)**	**0.77 (0.62–0.97)**	**0.79 (0.65–0.97)**
Facility Capacity Index	—	1.01 (0.99–1.02)	1.01 (0.99–1.02)	1.02 (1.00–1.03)
Maternal Severity Score	—	—	**1.94 (1.71–2.19)**	**1.94 (1.71–2.20)**
Human Development Index Group				
High/very high	—	—	—	Ref
Medium				**6.31 (2.53–15.74)**
Low				**4.86 (1.85–12.77)**

Abbreviations: CI, confidence interval; OR, odds ratio; SMO, severe maternal outcome.

^a^ Total SMO – 305 missing values.

^b^ Multivariate regression analyses. The multivariable analysis was performed using three different models: Model 1=Admission to ICU, age, marital status, level of Education, number of pregnancies, and Facility Capacity Index; Model 2=Admission to ICU, age, marital status, level of education, number of pregnancies, Facility Capacity Index, and Maternal Severity Score; and Model 3=Admission to ICU, age, marital status, level of education, number of pregnancies, Facility Capacity Index, Maternal Severity Score, and Human Development Index Group.

## 4 DISCUSSION

The present study demonstrated that admission to an ICU was independently associated with a significant reduction in the odds of maternal mortality in severely ill obstetric patients. When analyzing maternal mortality and admission to an ICU alone or within traditional variables associated with maternal mortality, there was no association between maternal death and admission to an ICU; however, when introducing the severity score in the model (MSS), the utilization of an ICU was associated with fewer maternal deaths.

According to the present findings, the provision of intensive care for severely ill women is associated with reduced maternal mortality, but this association may not be valid for pregnant women with complications from less severe conditions. It was demonstrated that those who benefit from an ICU are those with severe maternal conditions. It is not enough to provide intensive care to any woman with a complication; rather, it is necessary to make judicious use of resources and ensure intensive care services are available and provided to those who need it most.

The cross‐sectional study design and the pragmatic definition of maternal death (up to 7 days postpartum) used in the present study may be limitations for the results. In addition, the present analysis was not pre‐planned, which may introduce some bias in the results and the findings may not reflect countries with different health system profiles. Another limitation is that different criteria for admission to an ICU were used in each facility, including possible differences in obstetrics management, which may affect maternal outcomes. Nevertheless, this reflects the wide variation in access to an ICU for the obstetrical population.

The present rate of ICU utilization of 0.5% for the whole population of women giving birth was in accordance with the range suggested in the literature of studies in obstetric intensive care (0.5–6 per 1000 deliveries).^9^

In the present study, only 11.6% of women with maternal complications had access to intensive care. The availability of beds and the availability of an ICU in general were not able to be differentiated; admission to an ICU may not reflect access per se, as there are differences between facilities and countries in how ICUs are used, particularly admission criteria. The cost for the maintenance of an ICU is a barrier to the use of these services and has been discussed in other studies.^10^, ^12^

When considering only individual characteristics, higher level of education appears protective for maternal mortality. This is in accordance with previous publications showing a relationship between lower level of education and a higher likelihood of maternal death; less‐educated women present at a hospital later and in a more severe condition.^20^

Only 30% of women with SMO were admitted to intensive care, while 70% of maternal deaths occurred in women who did not use an ICU ‐ adequate use of the ICU is, therefore, an important issue. Even in facilities with ICU availability, there may be a lack of protocols for using the ICU among obstetric patients.^5^

Another factor influencing the occurrence of deaths among women not admitted to an ICU is the failure to recognize the severity of the disease, delay in referral, and low level of access to the healthcare system.^21^ The second and third delay may also be a contributory factor to the deaths occurring in the ICU as women may be admitted in such severe conditions that they may not be able to be saved.^21^ This is a complex issue that involves more than the relationship between supply and demand; it is influenced by factors such as financing and acceptance by women seeking care.^22^

There is an urgent need for health systems to correctly and quickly identify severely ill obstetric patients and prioritize their treatment according to severity, similar to the screening protocols routinely used in emergency departments to provide timely care to those in greatest need.^23^ When considering the severity of the clinical condition, the use of an ICU was associated with an approximately 80% reduction in the odds of maternal death. In other words, ICUs appear to make a significant difference for women who do need this level of care.^4^ Rather than having an ICU bed, knowing how to properly use it is critical for improving outcomes in obstetrical care.

The inclusion of the HDI in the model produced similar results, suggesting that the reduction of maternal mortality is associated with the use of an ICU according to the severity rating, even in low HDI settings. Thus, again, rather than the availability of ICU beds, the organization of maternity care in emergency rooms with standardization of criteria for the use of intensive care in order to provide equitable service is crucial for the efficiency of intensive care services in obstetrics.^12^, ^24^

The protective factor of ICUs shown in the present study resulted from the analysis of the risk of maternal death in a population with SMO. Therefore it is necessary to highlight that all women included in the models, by definition, already had the criteria of severity. Even among severe cases, some will not benefit from use of an ICU. That means that in addition to achieving efficient mechanisms to identify which women would benefit from the use of ICUs, there is a group of women presenting severity criteria who will not benefit from admission to an ICU at all. The results of the present study show that the central problem is not just “the use of ICU,” but who should use it.

For some women, the use of intermediate obstetric care units could provide the required attention according to the severity of the case, with less intensive care than that provided by an ICU, but more discerning than that found in common hospital wards.^25^ This strategy could reduce other problems when managing obstetric patients. Intermediate care units do not need critical care professionals and are less difficult to manage than an ICU; the insufficient number of ICU beds^25^ could then be more rationally used and, finally, the high costs of using an ICU^10^ can be more balanced with a more optimized use. This strategy of managing severe obstetric patients is described as a strategy to offer equitable care for obstetric patients with lower costs than the use of an ICU^5^, ^25^ and should be more deeply studied to improve maternal care.

## AUTHOR CONTRIBUTIONS

JPS conceived the idea and study design. OT, JGC, JPV, GT, and JPS participate in the data collection. FMS, OT, RCP, and JPS performed the analysis. FMS, OT, and RCP wrote the first draft. All authors contributed to the final version of the manuscript and accepted the final version.

## CONFLICTS OF INTEREST

The authors have no conflicts of interest.

5

**Table 4:** Distribution of countries participating in the WHOMCS according to the Human Development Index.

HDI category	Country	Hospitals	All women	Live births	Women with potentially life‐threatening conditions		Women with SMO		Maternal near‐miss cases		Maternal deaths	
		n	n	n	n	%	n	%	n	%	n	IHM MR
Low	Afghanistan	8	26 148	25 227	923	36.6	440	17.4	421	16.7	19	75
Low	Angola	20	10 450	9966	587	58.9	92	9.2	57	5.7	35	351
Low	Democratic Republic of Congo	21	8756	8395	501	59.7	115	13.7	88	10.5	27	322
Low	Kenya	20	20 354	19 658	1511	76.9	132	6.7	77	3.9	55	280
Low	Nepal	8	11 290	10 999	363	33.0	73	6.6	65	5.9	8	73
Low	Niger	11	11 116	10 714	516	48.2	223	20.8	196	18.3	27	252
Low	Nigeria	21	12 841	11 775	1701	144.5	371	31.5	298	25.3	73	620
Low	Pakistan	16	13 175	12 729	1158	91.0	132	10.4	94	7.4	38	299
Low	Uganda	21	10 923	10 467	691	66.0	152	14.5	120	11.5	32	306
Medium	Cambodia	5	4725	4635	288	62.1	64	13.8	59	12.7	5	108
Medium	China	21	13 277	13 242	927	70.0	34	2.6	34	2.6	0	0
Medium	India	21	31 318	30 094	2214	73.6	283	9.4	174	5.8	109	362
Medium	Jordan	1	1167	1158	119	102.8	5	4.3	5	4.3	0	0
Medium	Mongolia	5	7365	7303	823	112.7	62	8.5	61	8.4	1	14
Medium	Nicaragua	8	6571	6426	1707	265.6	125	19.5	119	18.5	6	93
Medium	Occupied Palestinian Territory	1	980	975	150	153.8	3	3.1	3	3.1	0	0
Medium	Paraguay	6	3610	3595	106	29.5	11	3.1	8	2.2	3	83
Medium	Philippines	13	10 783	10 609	830	78.2	41	3.9	29	2.7	12	113
Medium	Thailand	12	8973	8894	656	73.8	53	6.0	51	5.7	2	22
Medium	Vietnam	15	15 437	15 411	138	9.0	33	2.1	33	2.1	0	0
High	Brazil	7	7058	7019	505	71.9	18	2.6	17	2.4	1	14
High	Ecuador	18	10 245	10 108	1429	141.4	39	3.9	30	3.0	9	89
High	Lebanon	9	4044	4008	210	52.4	20	5.0	18	4.5	2	50
High	Mexico	14	13 309	13 167	1052	79.9	157	11.9	153	11.6	4	30
High	Peru	16	15 285	15 021	1116	74.3	175	11.7	169	11.3	6	40
High	Sri Lanka	14	18 129	17 988	862	47.9	76	4.2	73	4.1	3	17
Very high	Argentina	14	9807	9729	768	78.9	60	6.2	51	5.2	9	93
Very high	Japan	10	3537	3527	655	185.7	21	6.0	21	6.0	0	0
Very high	Qatar	1	3950	3932	509	129.5	14	3.6	14	3.6	0	0
	Overall	357	314 623	306 771	23 015	75.0	3024	9.9	2538	8.3	486	158

Abbreviations: IHM MR, Institute for Health Metrics mortality rate; SMO, severe maternal outcome; WHOMCS, WHO Multicountry Survey on Maternal and Newborn Health.

## Supporting information


**Appendix S1**. The Facility Capacity Index (FCI) for the WHO Multicountry Survey.
