# Supplementary material for: Provision of intensive care to severely ill pregnant women is associated with reduced mortality: Results from the WHO Multicountry Survey on Maternal and Newborn Health
Source: Int J Gynaecol Obstet. 2020 Jul 12;150(3):346–53. doi: 10.1002/ijgo.13241 (PMC7496974; doi:10.1002/ijgo.13241)
Supplement: Supplementary file 1 — Appendix S1. The Facility Capacity Index (FCI) for the WHO Multicountry Survey. [file IJGO-150-346-s001.doc]

**Appendix.** The Facility Capacity Index (FCI) for the WHO Multicountry Survey

The FCI is a proxy for the institution’s capacity to provide obstetric care, comprising six areas that reflect the standard of basic services, medical services, emergency obstetric services, laboratory tests, hospital practices, and human resources, calculated into a continuous index and categorized as “good,” “poor,” or “very poor.”

The FCI score is calculated by allocating 2 points to a facility for each of the 25 “essential” services and 1 point for each additional service available.

Scores range up to 59 points for each facility. Facilities with all essential services can reach 50 points.

| **Essential service** | **Additional service score** |
| --- | --- |
| **Standard of building / basic services** | |
| Electricity, water supply, and sewerage system |  |
| Generator |  |
| Refrigerator |  |
| Sterilization |  |
| Telephone |  |
| Ambulance |  |
| **Medical services** | |
| Blood bank | Adult ICU |
| Routine screening of donor blood (HIV, Hep B, syphilis) | Neonatal ICU or any other newborn care with indicators |
| Biochemical/clinical laboratories | High-risk pregnancy beds |
| **Emergency obstetric services** | |
| Administration of parenteral antibiotics |  |
| Administration of uterotonics (oxytocin, misoprostol, or other uterotonics) |  |
| Administration of magnesium sulphate |  |
| Manual removal of placenta |  |
| Removal of retained products |  |
| Vacuum extraction or forceps delivery |  |
| Blood transfusion |  |
| Hysterectomy |  |
| Neonatal resuscitation |  |
| **Laboratory tests** | |
| Hemoglobin | Bilirubin |
| Platelet count | Lactate |
| Coagulation tests | Blood gas analysis |
|  | Creatinine |
| **Hospital practices** | |
| WHO/local guidelines currently in use |  |
| Maternal death review |  |
| **Human resources** | |
| Obstetrician available 24/7, in the facility | Obstetrician available 24/7 on call |
| Anesthesiologist 24/7, in the facility | Anesthesiologist available 4/7 on call |
|  |  |
